# Biofilm Formation and Motility Depend on the Nature of the *Acinetobacter baumannii* Clinical Isolates

**DOI:** 10.3389/fpubh.2016.00105

**Published:** 2016-05-24

**Authors:** Saranya Vijayakumar, Sangeetha Rajenderan, Shakti Laishram, Shalini Anandan, Veeraraghavan Balaji, Indranil Biswas

**Affiliations:** ^1^Department of Clinical Microbiology, Christian Medical College, Vellore, India; ^2^Department of Microbiology, Molecular Genetics and Immunology, University of Kansas Medical Center, Kansas City, KS, USA

**Keywords:** *Acinetobacter baumannii*, biofilm, motility, multidrug resistance, India

## Abstract

*Acinetobacter baumannii* is a nosocomial pathogen involved in various infections ranging from minor soft-tissue infections to more severe infections such as ventilator-associated pneumonia and bacteremia. The severity and the type of infections depend on the genetic and phenotypic variations of the strains. In this study, we compared the extent of biofilm formation and motility displayed by 60 multidrug-resistant *A. baumannii* clinical strains isolated from blood and sputum samples from patients from Southern India. Our results showed that isolates from the sputum samples formed significantly more robust biofilm compared to the blood isolates. On the other hand, we observed that the blood isolates were more motile than the sputum isolates. To the best of our knowledge, this is the first study that systematically evaluated the correlation between these two phenotypic traits and the nature of the isolates.

## Introduction

*Acinetobacter baumannii* is a nosocomial pathogen that can cause a wide array of infections ranging from minor skin and soft-tissue infections to more severe invasive diseases, such as bacteremia, meningitis, and ventilator-associated pneumonia (VAP). VAP typifies serious hospital-acquired infections due to colonization of *A. baumannii* in the airway *via* environmental exposure. The mortality rate associated with *A. baumannii* induced VAP is between 40 and 70% (1, 2). The patients with the highest mortality tend to be older, immunocompromised, have prolonged intubation, and are at a greater risk of infection by other pathogens. In the intensive care setting, *A. baumannii* also causes serious bloodstream infections (3). The pathogen primarily enters into the bloodstream through lower respiratory tract infections and intravascular devices (4–7). Wound and urinary tract infections also lead to bloodstream infections (4). Like VAP, the risk factors for bloodstream infections include among others immunosuppression, colonization with *A. baumannii*, and invasive procedures (6–8). The mortality rates associated with the *A. baumannii* bloodstream infections ranges between 28 and 43%; however, the issue is highly debatable (3, 4, 9–11).

The pathogen’s ability to survive and to persist for extended periods of time on surfaces makes it a frequent cause for health-care-associated infections. Moreover, emergence and spread of multiple drug resistance (MDR) *A. baumannii* is an area of great clinical concern. *A. baumannii* is becoming resistant to most of the commonly used antibiotics, including aminoglycosides, broad-spectrum-β-lactams, and quinolones (12–16). MDR- or pandrug-resistant strains pose challenges to any clinician treating the infections caused by these strains. The drug resistance also imposes an additional economic burden on health-care systems (16, 17). There is an urgent need for the development of novel strategies to control infections caused by *A. baumannii*.

*Acinetobacter baumannii* encodes multiple virulence factors that contribute to the pathogenesis of this organism. Among them, the ability to form robust biofilm is one of the key virulence attributes of this pathogen. Formation of biofilm requires expression of the CsuA/BABCDE chaperon–usher complex required for the assembly and production of pili involved in adhesion to abiotic surfaces (18). It has been shown that inactivation of just the *csuE* gene eliminates pili production and biofilm formation. The *csu* operon is controlled by a two-component system, BfmRS, and the inactivation of BfmR abolishes expression of this operon and therefore pili and biofilm formation (19). Some strains of *A. baumannii* also produce relatively short pili that are CsuA/BABCD independent. These short pili are involved in the attachment to biotic surfaces, such as human respiratory cells (20), whereas other *A. baumannii* strains, such as 307-0294, encode a cell-surface-associated protein, Bap, which is homologous to a staphylococcal protein, is important for the stabilization of mature biofilm on abiotic and biotic surfaces (21, 22). The cell-surface-associated protein OmpA also plays an important role in biofilm formation (23). *A. baumannii* secretes an extracellular polysaccharide poly-β-(1, 6)-*N*-acetylglucosamine (PNAG) that functions as an intracellular adhesion among the biofilm-associated cells (24). *A. baumannii* has the ability to survive prolonged exposure to dry conditions and nutrient limiting environments (20, 25–28). This survival trait allows the organism to persist on the abiotic surfaces that are present in the health-care setting. The extraordinary survival ability has also been implicated to resistance to various antibiotics and desiccation (18, 26). Furthermore, it has also been proposed that the resistance phenotypes of the clinical isolates could be attributed to the ability form biofilm on abiotic surfaces, particularly the isolates from patient inserts (18, 29, 30). A recent study by Espinal and colleagues suggested that the clinical isolates that form higher biofilms tolerate and survive desiccation better than the non-biofilm forming clinical isolates (28).

*Acinetobacter baumannii* lacks flagella and has been described as non-motile (18, 31). Recent whole genome sequence analysis has also confirmed the absence of flagellar genes in *A. baumannii* suggesting the lack of true swarming motility, which requires flagella (32). However, several recent studies have demonstrated that *A. baumannii* displays twitching motility that allows the organism to spread rapidly on semisolid and certain abiotic surfaces (32–36). Twitching motility is mediated by type IV pili by the action of extension and retraction of the pili. The genes necessary for the assembly of type IV pili (*pilA-C*, *pilF*, *pilM-Q*, *pilW*, *pilZ*), twitching (*pilR-T*), and the pilin filament (*pilA*) are all present in the *A. baumannii* genome. Two groups have recently shown that type IV pili are necessary for both surface and twitching motility (32, 36). Furthermore, analysis of genome sequences suggests the presence of multiple type IV pili-associated genes in all the *A. baumannii* strains whose complete genome information is available. Moreover, a positive correlation between the PilA encoding gene and the degree of twitching motility has been demonstrated in clinical isolates (33). The motility in bacteria is regulated by multiple signal transduction pathways (37). Several environmental factors, such as light (particularly in the blue wavelength), iron availability, and stress, will influence the motility in *A. baumannii* (34, 38).

Although most of the studies involving clinical isolates focused on the biofilm formation or drug resistance, there is no systematic study to correlate between nature of the isolate with the biofilm and motility. In this study, we used 60 multidrug resistant clinical isolates that are from sputum and blood samples. We first determined the clonal lineage of these isolates and evaluated the synergistic activity of sulbactam with meropenem or colistin. We then investigated the capacity to form biofilm on polystyrene tubes and the twitching and surface motility of these clinical isolates. We found that sputum isolates tend to form more biofilm as compared to the blood isolates. On the other hand, blood isolates displayed more motility than the sputum isolates. This is the first systematic study to delineate the two important phenotypic traits with the origin of clinical isolates.

## Materials and Methods

### Bacterial Isolates and Growth Conditions

This study was conducted between January 2014 and June 2014 at Christian Medical College (CMC), Vellore, India. The 60 isolates investigated were obtained from the respiratory secretions, and blood samples of 60 patients attending different outpatient wards. The blood and the sputum isolates were from different cohort of patients. The protocol was reviewed by the Institutional Review Board (IRB), CMC, Vellore, and determined to meet the necessary criteria for exemption since the project falls under the category of observational study. Per local policies and through consultation with the IRB, written patient consent was not required and formal ethical approval was reviewed and waived.

The samples were sent to the Clinical Microbiology Department for further analysis. Gram-negative bacilli isolates were further identified as *A. baumannii – Acinetobacter calcoaceticus* complex and were confirmed by biochemical tests based on carbohydrate and amino acid utilization. Antimicrobial susceptibility was tested for all the isolates on Mueller–Hinton agar (BD), using the standard Kirby–Bauer disk diffusion method according to the guidelines of the Clinical and Laboratory Standards Institute (CLSI). The following antimicrobials were tested: amikacin (10 μg), aztreonam (10 μg), ciprofloxacin (10 μg), ceftazidime (10 μg), cefepime (10 μg), gentamicin (10 μg), imipenem (10 μg), meropenem (10 μg), piperacillin-tazobactam (10 μg), tobramycin (10 μg), and trimethoprim–sulfametoxazol (30 μg).

### Metallo β-Lactamase Detection

A freshly prepared bacterial suspension adjusted to 0.5 McFarland unit (1.5 × 10^7^ cells) was streaked for confluent growth on a Mueller–Hinton agar plate using a swab. Five microliters of EDTA (0.35M EDTA) solution were added into a paper disk (6 mm diameter) and dried without overflowing. The disks were placed at the center of the plate. Ten micrograms of meropenem, meropenem with EDTA, cefepime, and cefepime with EDTA disks were placed at a distance of 10 mm from the center, and the plate was incubated at 37°C for 16–18 h. Disks containing EDTA alone served as the negative control. The appearance of zone around the antibiotics containing EDTA disks would indicate a metallo β-lactamase (MBL) producer. We consider an isolate to be MBL-positive if the zone of inhibition is larger than 2 mm when EDTA is added to the meropenem and cefeprime disks. The test was repeated at least three times.

### Biofilm Assay

Polystyrene (12 mm × 75 mm) tubes containing 1.5 ml of Mueller–Hinton broth was inoculated with 30 μl of an overnight liquid culture, and the tubes were incubated at 37°C for 48 h. The liquid media was discarded, and the adherent cells were washed twice with phosphate-buffered saline (PBS) and stained with 0.02% of crystal violet for 10 min. The stain was eluted from the adherent cells using an ethanol:acetone (1:5) solvent and vortexing for 5 min. Absorbance of the eluted solvent was measured, after diluting 10-fold with the solvent, at 580 nm using an UV visible spectrophotometer (UV-1601, SHIMADZU). The assay was done at least three times using fresh samples each time.

### Motility Assay

Modified LB broth (tryptone – 10 g/l; NaCl – 5 g/l; yeast extract – 5 g/l) with either 0.4 or 0.8% agar was used for all the motility assays. Freshly grown cultures were stabbed to enable spread of bacteria on the surface of the medium (0.4% semisolid) for swarming motility and the interphase between the bottom of the Petri dish and medium (0.8% semisolid) for twitching motility, as described previously (32). Plates were prepared on the same day as the inoculation. After inoculation, the plates were sealed with parafilm and incubated at 37°C for 48 h. Swarming positive isolates were defined as those strains that showed a zone of >10 mm around the site of inoculation. For twitching motility, the agar was discarded, and the plates were stained with 0.2% crystal violet before visualization and photographed. For each isolate, assays were performed at least three times.

### Antibiotic Sensitivity Assays

A microbroth dilution assay was used to determine MICs for sulbactam, meropenem, and colistin as per Clinical Laboratory Standard Institute guidelines. Checkerboard synergy was performed and calculated using this formula. Fractional inhibitory concentrations (FICs) indices were calculated as (MIC of drug A or B in combination)/(MIC of drug A or B alone), and the FIC index was obtained by adding the FIC values. FIC indices were interpreted as synergistic if values were <0.5, additive or indifferent if 0.5–4.0 and antagonistic if >4.0. Time-kill assay was performed at ½ the MIC value for each drug. An antimicrobial solution at the required concentration was prepared in cation-adjusted Mueller–Hinton broth at a final volume of 10 ml. An inoculum of approximately 6 × 10^5^ was inoculated and incubated at 37°C for 24 h. CFU per milliliter was determined at 0, 3, 6, and 24 h of incubation. For determining CFU per milliliter of the organism, 0.1 ml aliquots from each tube were transferred to 10 ml normal saline, serially diluted, and plated onto nutrient agar in duplicates. Counts were obtained by multiplying the average number of colonies from the duplicate plates by the dilution factor. Synergy was interpreted as more than 2 log_10_ reductions in the CFU per milliliter in the tube containing both drugs compared to the most active single agent.

### Statistical Methods

Frequency distribution was done for categorical variables and descriptive statistics such as mean, median, SD, and minimum and maximum for continuous variables. Independent sample *t*-test was used to find the difference in the biofilm between the blood and the sputum isolates. Association between motility status (NM/IM/HM) and the isolates (blood/sputum) was calculated using chi square test. Histogram, error plot, and clustered bar chart were also used for data presentation.

## Results

In this study, we employed 60 clinical *A. baumannii* strains isolated from 60 different patients from a tertiary care facility in Southern India (Tamil Nadu). The age of the patients varied from newborn to 74 years and roughly 30% were female patients. Among the isolates, half of them were from sputum samples and the other half were isolated from the blood cultures. All the isolates displayed resistance to multiple antibiotics including broad-spectrum-β-lactams, such as cefepime, imipenem, and meropenem (data not shown). We then investigated the clonal groupings among the isolates by determining the presence of *bla_*OXA-51-like*_*, *csuE*, and *ompA* allelic variants using Group 1 PCR, as described by Turton and colleagues (39). We found that 21 (70%) sputum isolates and 17 (57%) blood isolates showed characteristic amplification patterns of European clonal group II (ECII, Table 1). Only one sputum isolate and two blood isolates displayed a PCR profile similar to European clonal group III (ECIII, Table 1). The rest of isolates generated patterns that were outside the pan-European clonal lineages I-III. Based on the PCR amplification pattern, these non-European clones can be divided into four categories: UC-I through -IV (Table 1). We observed that sputum samples are less diverse, containing only the UC-II (only *ompA* and *bla_*OXA-51-like*_* amplification) and UC-IV groups (only *bla_*OXA-51-like*_* amplification). On the other hand, blood isolates were more diverse containing all four UC types.

**Table 1:** **Phenotypic properties of the (A) blood isolates and (B) respiratory isolates**.

Sample ID	Age/sex	Clone group^#^	Biofilm*	Motility^@^ (mm)	MIC^$^			Synergy^+^	
					MER	SUL	COL	M + S	C + S
**(A) Phenotypic properties of the blood isolates**									
B21029^E^	25F^T^	EC-II^(OEX)^	1.61 ± 0.09	10	128	64	0.25	IND^a*^	IND^b*^
B10883^E^	4F	UC-I	1.95 ± 0.18	>40	64	64	1.0	IND^a*^	IND^c*^
B29276	0M^IV^	EC-II^(OEX)^	2.08 ± 0.85	20	16	32	0.5	IND^b^	IND
B25330	0M^T^	EC-III^(EX)^	2.20 ± 0.71	25	64	64	0.5	IND^b*^	IND^c*^
B28412	0M^T^	UC-II^(OX)^	1.01 ± 0.68	<5	32	32	0.5	SYN^b*^	IND^c*^
B28641^E^	74M^IV^	UC-III^(X)^	2.68 ± 0.30	30	64	64	0.5	SYN^a*^	SYN^b*^
B13833^E^	75M^IV^	UC-IV^(O)^	1.03 ± 0.14	35	32	32	0.5	SYN^b*^	IND^c*^
B1301	54F^T,IV^	UC-I	2.58 ± 0.20	35	128	128	0.5	SYN^a^	IND^b,c*^
B29819	25M	EC-II^(OEX)^	2.11 ± 0.09	20	128	128	0.5	SYN^b*^	SYN^c*^
B11439^E^	45F^T^	EC-II^(OEX)^	3.11 ± 0.35	10	64	32	0.5	IND^b*^	SYN^b*^
B27768	50M^T^	EC-II^(OEX)^	0.73 ± 0.08	10	128	32	0.5	IND^a*^	IND^c*^
B10631^E^	13M^IV^	UC-II^(OX)^	2.06 ± 0.23	10	64	64	0.5	IND^b*^	IND^b*^
B4609^E^	13F^T,IV^	EC-II^(OEX)^	2.15 ± 0.20	25	16	32	0.5	SYN^b*^	IND^c*^
B27683^E^	2M	UC-V^(EO)^	3.00 ± 0.48	20	64	32	0.5	SYN^a*^	IND^c*^
B29467	65M	EC-III^(EX)^	2.43 ± 0.40	>40	64	32	0.5	SYN^b*^	SYN^c*^
B27919^E^	34M^T,IV^	EC-II^(OEX)^	2.45 ± 0.01	10	64	64	0.5	IND^a*^	IND^c*^
B28414^E^	74M^IV^	EC-II^(OEX)^	2.25 ± 0.15	<5	64	64	1.0	SYN^b*^	IND^b*^
B23356	9M^T,IV^	UC-IV^(O)^	1.23 ± 0.18	>40	64	32	1.0	IND^a^	IND^c*^
B8689	41M^T,IV^	UC-IV^(O)^	0.73 ± 0.02	>40	128	128	0.5	SYN^b*^	IND^a*^
B17606^E^	14M^T,IV^	EC-II^(OEX)^	1.97 ± 0.00	10	64	128	0.5	IND^a*^	IND^c*^
B8716^E^	1M	EC-II^(OEX)^	1.61 ± 0.45	10	128	64	0.25	IND^a^	IND^c*^
B8765	45M^T^	UC-II^(OX)^	1.24 ± 0.22	<5	256	64	0.5	IND^a*^	IND^c*^
B11911^E/N^	45M^IV^	UC-I	2.44 ± 0.78	10	256	64	0.5	IND	SYN
B5534	21F^T^	EC-II^(OEX)^	3.86 ± 0.89	>40	256	64	1.0	SYN^b*^	IND^c*^
B5208^E/N^	47M	EC-II^(OEX)^	3.09 ± 0.14	25	128	64	0.5	SYN^a*^	SYN^b*^
B5586	2F^T,IV^	EC-II^(OEX)^	2.03 ± 0.20	>40	512	128	1.0	IND^a*^	IND^c*^
B5868	17M^T^	EC-II^(OEX)^	1.46 ± 0.41	10	256	64	0.5	SYN^b*^	IND^c*^
B7370^E^	24F	EC-II^(OEX)^	1.46 ± 0.10	10	256	64	0.5	SYN^a*^	SYN^c*^
B8240^E^	58M	EC-II^(OEX)^	2.10 ± 0.37	15	128	64	0.5	IND^a^	IND^c^
B9464	24F^IV^	EC-II^(OEX)^	1.83 ± 0.09	<5	256	64	1.0	SYN	IND
**(B) Phenotypic properties of the respiratory isolates**									
SP344	18F^IV^	UC-II^(OX)^	1.78 ± 0.11	20	64	128	0.25	SYN^b*^	IND^c*^
SP858	25M^T^	UC-II^(OX)^	1.37 ± 0.07	<5	128	0.25	0.25	SYN^b*^	IND^b*^
SP1397^E^	48M^T^	EC-II^(OCX)^	3.43 ± 0.11	<5	512	128	0.25	IND^b*^	IND^c*^
SP1115^E^	62M	EC-II^(OCX)^	4.72 ± 0.44	<5^SM^	128	64	0.25	SYN^b*^	IND^c*^
SP1394	61F^T,IV^	EC-II^(OCX)^	3.82 ± 0.31	10	64	64	0.25	SYN^b*^	IND^c*^
SP1182	60M^T^	EC-II^(OCX)^	4.71 ± 0.25	10	128	64	0.25	IND^a*^	SYN^c*^
SP1974	57F^T,IV^	EC-II^(OCX)^	3.67 ± 0.08	<5	256	256	1	IND^a*^	SYN^c*^
SP1128	35M^T^	EC-III^(CX)^	3.59 ± 0.16	<5	256	128	0.5	SYN^a*^	IND^c*^
SP1977	61M^T,IV^	EC-II^(OCX)^	5.01 ± 0.70	10	256	512	0.5	SYN^b^	SYN
SP1054	39F^T,IV^	EC-II^(OCX)^	3.90 ± 0.10	<5^SM^	64	64	1	SYN^a^	IND^c*^
SP1917^E/N^	68M^T,IV^	EC-II^(OEX)^	3.62 ± 0.16	<5	512	256	0.25	SYN^a*^	IND^b*^
SP1209	49 M^T,IV^	EC-II^(OCX)^	5.51 ± 0.63	<5^SM^	64	64	0.5	SYN^b*^	SYN^c*^
SP1201^E^	55M	EC-II^(OCX)^	3.72 ± 0.12	10	256	32	0.5	IND^a*^	IND^b*^
SP932^E^	52M	EC-II^(OCX)^	3.19 ± 0.12	10	256	64	1	SYN^a*^	IND^c*^
SP2006^E^	21M^IV^	UC-IV^(O)^	1.39 ± 0.09	<5	32	64	8	IND	SYN
SP443^E^	59M	UC-II^(OX)^	1.39 ± 0.00	<5	32	32	0.5	SYN^a^	IND^c*^
SP1022^E^	65M^IV^	EC-II^(OCX)^	2.44 ± 0.35	<5	256	64	1	SYN^b^	IND^c*^
SP1026^E^	28M^T^	EC-II^(OCX)^	3.95 ± 0.33	<5	256	32	1	SYN^a*^	IND^c*^
SP3179	29M^T,IV^	EC-II^(OCX)^	3.70 ± 0.17	<5	128	64	1	IND^b*^	IND^b,c*^
SP2200	40M^T,IV^	UC-II^(OX)^	2.58 ± 0.30	<5	32	16	1	SYN^b*^	IND^c*^
SP1950	68M^T^	EC-II^(OCX)^	3.35 ± 0.58	<5	256	256	0.5	SYN^b*^	IND^c*^
SP715	17M^T^	UC-II^(OX)^	3.02 ± 0.63	<5	256	32	0.25	SYN^b*^	SYN^c*^
SP1909^E/N^	28F	EC-II^(OCX)^	3.50 ± 0.04	10	512	512	0.5	IND	SYN
SP1306^E^	47F^IV^	UC-II^(OX)^	1.67 ± 0.03	<5^SM^	32	64	1	IND^b^	IND^c*^
SP1766^E^	40M^T^	EC-II^(OCX)^	6.78 ± 0.33	>40^SM^	512	64	0.5	SYN^a*^	SYN
SP1834^E^	50M	EC-II^(OCX)^	3.08 ± 0.28	<5	256	256	0.5	SYN	SYN
SP1840^E^	75M	EC-II^(OCX)^	6.39 ± 0.47	>40^SM^	512	128	0.5	SYN^a*^	IND
SP1851^E/N^	48M^T,IV^	EC-II^(OCX)^	4.87 ± 0.93	<5	512	256	1	IND	IND
SP1843^E/N^	36F	EC-II^(OCX)^	2.89 ± 0.10	<5	256	128	0.5	SYN^b*^	IND
SP1770	24M^T^	UC-II^(OX)^	2.14 ± 0.08	<5	128	128	0.5	IND^a^	SYN

*^#^Clonal grouping done based on Turton et al. (39). EC, European clone; UC, unknown clone*.

*The alleles used for clone determination are O, OmpA; E, CsuE, X, Oxa*.

*OD_580_ values of the crystal violet stain eluted from the biofilm biomass grown on polystyrene tubes.

*^@^Twitching motility scored on Petri plates after staining with crystal violet. Diameter of zone of migration is shown*.

*^$^MIC determined by microdilution broth method*.

*^+^Determined by time-kill assay*.

*IND = Indifferent; SYN = Synergy*.

*MER and M = Meropenem; SUL and S = Sulbactam; COL and C = Colistin*.

*E: MBL-positive as determined by EDTA plate assay*.

*N: NDM positive isolates as determined by PCR analysis*.

*Synergism was determined by time-kill assay only*.

Explanation for a/a*, b/b*, and c/c* are: a – meropenem, a* – meropenem positive for bactericidal effect; b – sulbactam, b* – sulbactam positive for bactericidal effect; c – colistin, c* – colistin positive for bactericidal effect.

*T, intubated; IV, intravenous lines; SM, swarming motility*.

To understand more about the MDR properties of these isolates, we first verified whether MBLs are involved in the resistance toward β-lactam antibiotics. For this, we performed disk diffusion assays using EDTA to distinguish MBL producing isolates, as described previously (40). We found that 16 isolates from sputum and 16 isolates from blood were MBL-positive (Table 1). We then checked the presence of New Delhi metallo-β-lactamse (NDM-1) among the isolates by PCR. We found two blood isolates (B11911 and B5208) and four sputum isolates were NDM-1 positive (SP1917, SP1909, SP1851, and SP1843). Among the two blood isolates, one belongs to EC-II (B5208) and the other to UC-I (B11911). On the other hand, all sputum isolates were from the clonal lineage EC-II. With the rest of the MBL-positive isolates, we also checked for the presence of VIM-1 and found none of the isolates were positive for VIM-1. Thus, it is possible that the rest of the MBL-positive isolates encode other types of MBL such as IMP or SIM. We have recently determined the complete genome sequence of B11911 and SP1917 isolates, and we found that both the isolates encode the *bla*NDM-1 gene (41).

Since these isolates were all MDR positive, we then decided to determine synergy between sulbactam with either meropenem or colistin. For this test, we first determined the MIC_50_ values for the isolates and found that the values are 0.5, 64, and 128 μg/ml for colistin, sulbactam, and meropenem, respectively. We then determined the antibiotic synergy using a time-kill assay. For the blood isolates, the sulbactam and meropenem combination resulted in synergy on 50% isolates with 82% showing bactericidal activity. The sulbactam and colistin combination yielded relatively low synergy with only 17% isolates displaying synergy and 96% were bactericidal. On the other hand, among the sputum isolates, drug synergy was seen among 76% of the isolates when the sulbactam and meropenem combination was used. Like in blood isolates, synergy was seen only among 15% isolates when the sulbactam and colistin combination was used. With both types of isolates, we did not observe any antagonistic activity with both the antibiotic combinations. Taken together, our results indicate that the sulbactam and meropenem combination could be an effective alternative treatment strategy for MDR *A. baumannii* infections, while the sulbactam and colisitin combination will not be very effective.

**Figure 1:**
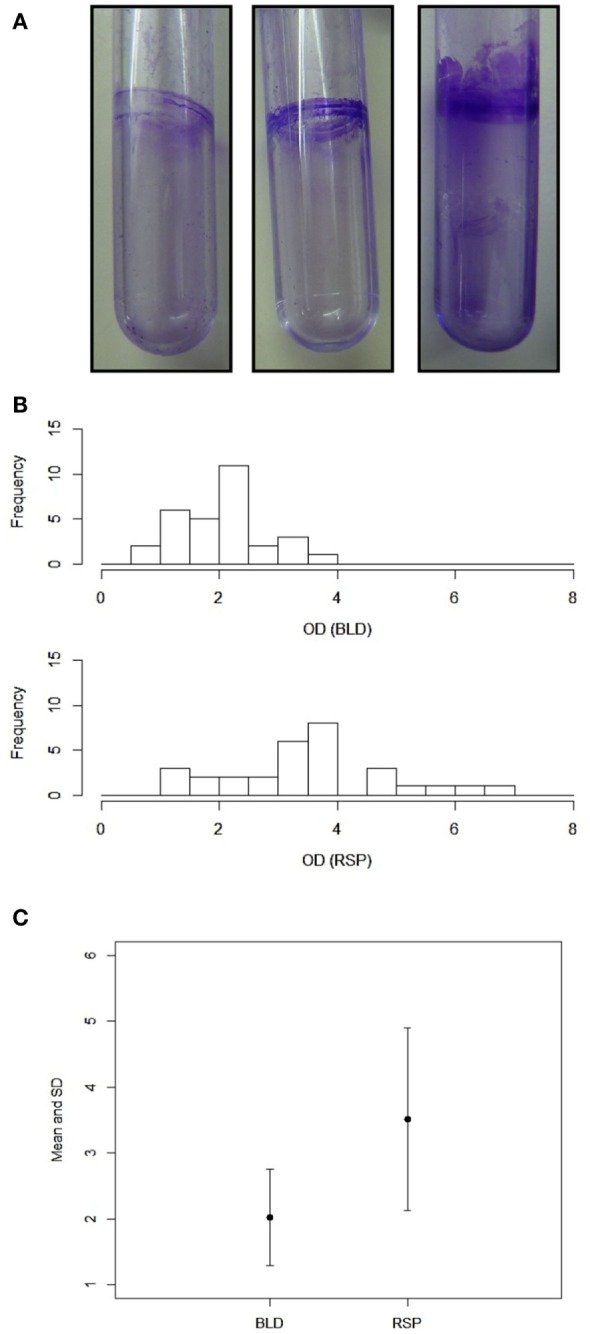
**Biofilm formation by *A. baumannii* clinical isolates**. **(A)** Representative polystyrene tubes with varying degrees of biofilms. Biofilms were stained with crystal violet after 2 days of incubation. **(B)** Quantitation of biofilm mass by crystal violet. Bar diagrams showing OD values (*x*-axis) against the number of isolates (*y*-axis). **(C)** Statistical analysis of the blood and respiratory isolates (*p* < 0.001). Values for mean and SDs are shown. Representations: BLD, blood isolates; RSP, respiratory isolates. Experiments were repeated at least three times.

One of most important virulence-related attributes of *A. baumannii* is the ability to form biofilm. Therefore, we decided to measure the biofilm forming capacity of these two groups of isolates. Since *A. baumannii* is able to form biofilm on polystyrene surfaces (33), we used polystyrene test tubes as an abiotic surface for the biofilm growth (Figure 1A). We found that most of the isolates from both blood and sputum were able to form varying degrees of biofilm on polystyrene. We found that blood isolates formed less robust biofilm compared to the sputum isolates, which formed thicker biofilm. The OD_580_ values for the blood isolates varied between 0.73 (B8689) to 3.86 (B5534) with majority of the isolates yielding values <2.0. On the other hand, the OD_580_ values for the sputum isolates were between 1.39 (SP443) and 6.78 (SP1766), with majority of the isolates yielding values above 2.0 (Figure 1B). The difference in the biofilm forming capacity was statistically significantly higher (*p* < 0.001; paired Student’s *t*-test) in the sputum isolates as compared to the blood isolates (3.51 ± 1.38 and 2.02 ± 0.73, respectively; Figure 1C). We found no correlation between the biofilm forming capacity and the MDR phenotypes (*p* < 0.5). However, the two sputum isolates (SP1766 and SP1840) that displayed the highest biofilm masses also showed the highest MIC_50_ values for meropenem (512 μg). Interestingly, these two isolates also formed pellicle during growth in liquid media; none of the other isolates formed pellicle. We did not observe any correlation between the clonality of the isolates and the biofilm forming capacity.

**Figure 2:**
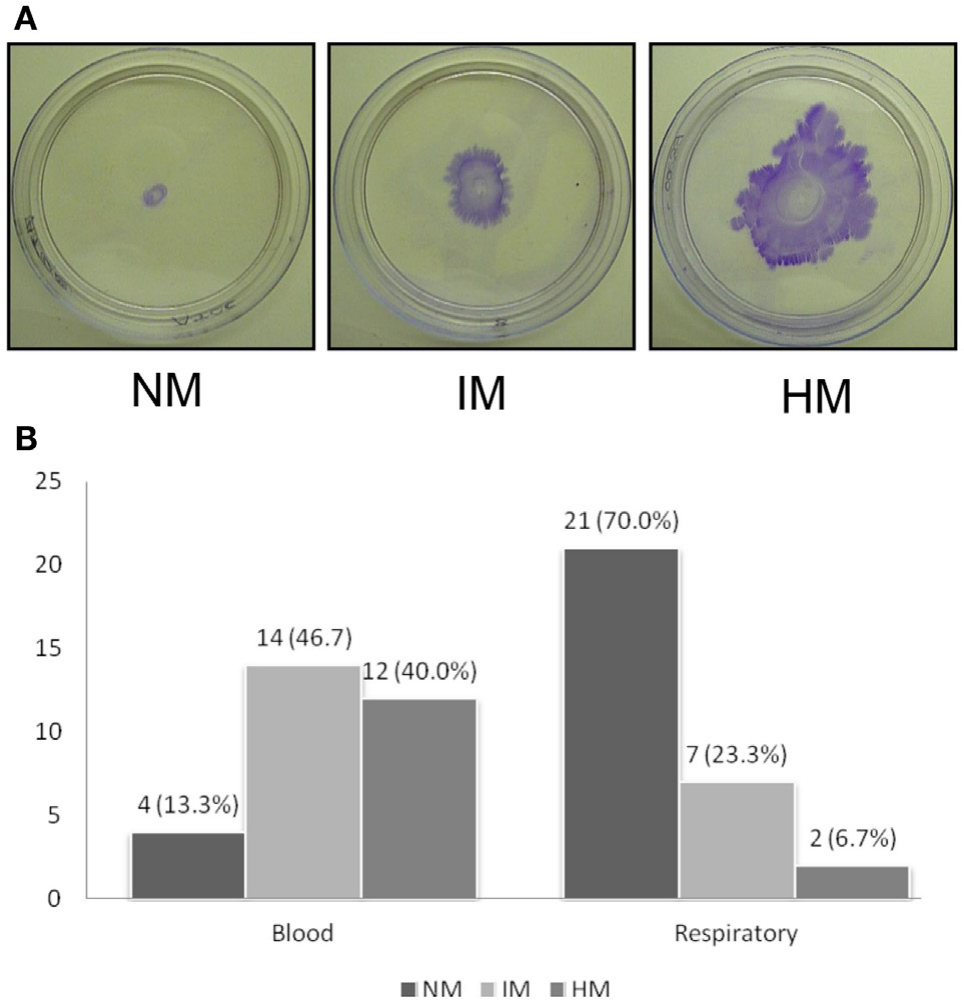
**Twitching motility displayed by *A. baumannii* clinical isolates**. **(A)** Assays were done using polystyrene Petri dishes by depositing inoculum at the interphase between the bottom of the Petri dish and agar. Plates were incubated for 48 h and stained with crystal violet after discarding the media. The average diameter of the zone of twitching was determined, and the isolates are classified as non-motile (NM, <5 mm), intermediately motile (IM, 5–20 mm), and highly motile (HM, >20 mm) are shown. Representative plates are shown. **(B)** Bar diagram showing number of isolates (*X*-axis) displaying NM, IM, and HM phenotypes. Experiments were repeated four or more times.

Because we found a significant difference in biofilm forming capacity, we decided to measure the motility among the isolates. We first measured the twitching motility displayed by the isolates. Twitching motility was assayed based on the ability of the cells to spread on the polystyrene Petri dishes. We found that the isolates displayed varying degrees of twitching motility. We categorized these isolates into three groups. If the twitching zone diameter was <5 mm, the isolate is considered as twitching negative. A twitching zone diameter between 5 and 20 mm is considered as intermediate while >20 mm of twitching zone was considered as highly motile isolate. As shown in Figure 2, the blood isolates were much more proficient in twitching motility as compared to the sputum isolates, which is statistically significant (*p* < 0.001). Twenty-six out of 30 blood isolates were positive for twitching motility with 12 isolates considered as highly motile (Figure 2B). Moreover, 6 of the 12 highly motile isolates displayed a zone of migration diameter higher than 40 mm. However, none of the twitching motility efficient blood isolates displayed any swarming-like motility on the semisolid plates containing 0.4% agar (data not shown). In contrast, less numbers of the sputum isolates were motile, only seven isolates displayed intermediate degree of motility, and only two isolates were in the highly motile category. Interestingly, the two highly motile isolates also formed the highest amounts of biofilm mass and pellicle (Table 1) despite the fact that growth of these isolates in liquid was similar to others. These two isolates also displayed swarming-like motility. Surprisingly, we also observed that some of the sputum isolates that were not efficient in twitching motility displayed swarming-like motility (Table 1). Notably, isolates SP1115, SP1054, and SP1306 were positive for swarming-like motility. Taken together, our results showed that blood isolates are much more motile compared to the sputum isolates.

## Discussion

Clearly, our current study sheds some light on the two important aspects of *A. baumannii* pathogenesis, the ability to form biofilm and motility. Although several studies have been performed to assay biofilm formation by clinical isolates, none of the studies systematically measured or compared biofilm formation in sputum against blood isolates. This is the first study to find that sputum isolates are mostly non-motile while the blood isolates are highly motile. In *A. baumannii*, several factors determine the biofilm-forming capacity. The chaperon/usher system and the OmpA protein are the two most important ones. The *csuE* and *ompA* genes are also used in determining the clonal groups of *A. baumannii* isolates. We observed that the sputum isolates belonging to the clonal lineage II formed robust biofilm compared to the rest of the isolates. Therefore, it is possible that the chaperon/usher system positively contributes to the biofilm formation in the clonal lineage II. However, we did not find any correlation between the clonal lineage and biofilm formation among the blood isolates. Overall, blood isolates formed less robust biofilm compared to the sputum isolates. Although we did not find any correlation between the clonal lineage and the biofilm forming capacity among the blood isolates, we did observe that the three isolates forming the most biofilm biomass all belong to clonal lineage II. Since we obtained *ompA* amplification for all the isolates except four during clonal lineage determination, it is possible that OmpA does not play a major role in biofilm formation at least in the blood isolates. Alternatively, the genes required for biofilm formation are either downregulated or not expressed in the blood isolates compared to the sputum isolates.

Among all the isolates, we found that only two isolates produced thick pellicles, whereas a few others produced very thin pellicles. Pellicles are bacterial masses arising at the interface between air and liquid during growth. Pellicle formation has been studied in many bacteria including *Bacillus subtilis*, *Pseudomonas aeruginosa*, *Shewanella oneidensis*, and *Vibrio parahaemolyticus* (42–46). Pellicle formation by the clinical isolates of *A. baumannii* has been recently studied (47, 48). In one study, about 50 proteins were found to be differentially expressed in the pellicle state (47). It appears that different types of pili, other than *csu* or type IV, are required for bacterial attachment and to maintain the entire mass floating on the top of the liquid medium. These different pili systems could also contribute to *A. baumannii* persistence in hospital settings. We are currently investigating the two respiratory isolates that produced maximum pellicle for persistence.

While biofilm-related phenotypes have been studied in *A. baumannii*, very little is known about the motility-related phenotypes. To our surprise, we found that the blood isolates are more frequently motile compared to the respiratory isolates. Motility requires the presence of type IV pili, and it has been shown that biofilm-forming cells often downregulate genes related to motility in other bacteria (49, 50). Type IV pilus biogenesis is not well studied in *A. baumannii*. In *Pseudomonas* spp., nearly 50 genes are involved in the regulation and biogenesis of type IV pili (51, 52). We speculate that the reason behind the blood isolates displaying high motility is due to over expression of type IV pili-related genes as compared to the sputum isolates. Alternatively, respiratory isolates that display little or no motility might be lacking type IV biogenesis genes. Recent comparative genomic studies have confirmed certain strain-specific variations in the type IV pili encoding genes among various *A. baumannii* isolates (53). Furthermore, Eijkelkamp and colleagues have recently shown that the gene encoding the major pilin subunit, *pilA*, is highly variable among the *A. baumannii* clinical isolates (33). Thus, it is possible that *pilA* alleles determine the degree of motility.

We found that only six isolates, all from respiratory samples, displayed swarming-like motility (Table 1). A recent study claimed that nearly all the clinical isolates from geographically diverse locations displayed swarming-like motility on 0.5% agarose-containing media (54). However, another study reported that swarming-like motility is about 8%, similar to what we found (10% considering both blood and respiratory isolates). There are several reasons that can account for this apparent discrepancy, including experimental conditions (media and the matrix), source and the nature of the isolates, and other external factors. Furthermore, both swarming-like and twitching motility are regulated by various environmental factors including stress, light, and temperature (32, 34, 55, 56). Thus, a slight variation of any of these physical factors could have a huge affect on both types of motility.

The exact reasons why respiratory isolates frequently form more biofilm and are less motile are currently unknown. We speculate that *A. baumannii* strains need to attach firmly to the alveolar cells, so that they can invade the host easily. The more motile isolates will not have sufficient time for invasion. Motility also requires synthesis of 1,3-diaminopropane (DAP), a polyamine produced by *A. baumannii* that is required for motility (54). It is possible that oxygen-rich environments suppress the production of DAP and thus the motility. The oxygen-rich environment also generates reactive oxygen species (ROS), and a recent study suggests that super oxide dismutase (SOD) is required for *A. baumannii* motility (56). Thus, it is also possible that ROS might inhibit biogenesis of type IV pili and other factors required for motility. We are currently studying the role of oxygen in *A. baumannii* motility with a few isolates.

In conclusion, this is the first systematic study involving two types of *A. baumannii* clinical isolates, blood and respiratory, to correlate with the capacity to form biofilm and motility traits. Our results showed that respiratory isolates form robust biofilm but are less motile while the blood isolates are more motile. However, we did not observe any correlation between the MDR phenotypes and motility or biofilm formation. Since motility was very frequent among the blood isolates, mechanistic investigation in motility would provide novel therapeutic strategies and control of the persistence of this pathogen.

## Author Contributions

SV: data collection and analysis, review of manuscript; SR: data collection and analysis, review of manuscript; SL: research and study design, review of manuscript; SA: research and study design, review of manuscript; VB: research and study design, review of manuscript; IB: research and study design, data collection and analysis, preparation and review of manuscript.

## Conflict of Interest Statement

The authors declare that the research was conducted in the absence of any commercial or financial relationships that could be construed as a potential conflict of interest.
